# QCM-Based HCl Gas Sensors Using Spin-Coated Aminated Polystyrene Colloids

**DOI:** 10.3390/polym12071591

**Published:** 2020-07-17

**Authors:** Young-Jae Jin, Jinyoung Park

**Affiliations:** 1 School of Applied Chemical Engineering, Kyungpook National University, 80 Daehak-ro, Buk-gu, Daegu 41566, Korea; lastlast7@naver.com; 2 Department of Polymer Science & Engineering, Kyungpook National University, 80 Daehak-ro, Buk-gu, Daegu 41566, Korea

**Keywords:** PS colloidal bead, amination, HCl gas, QCM, sensitivity

## Abstract

Hydrogen chloride (HCl) gas is highly toxic to the human body. Therefore, HCl gas detection sensors should be installed at workplaces where trace HCl gas is continuously generated. Even though various polymer-based HCl-gas-sensing films have been developed, simpler and novel sensing platforms should be developed to ensure the cost effectiveness and reusability of the sensing platforms. Therefore, we present a simple strategy to fabricate reusable HCl-gas-sensing platforms using aminated polystyrene (a-PS) colloids and investigate their sensitivity, reusability, and selectivity using a quartz crystal microbalance (QCM). The reusable *a*-PS(1.0) colloidal sensor with a high degree of amination (DA) exhibited the highest binding capacity (102 μg/mg) based on the frequency change (Δ*f*) during the HCl gas adsorption process. Further, its sensitivity and limit of detection (LOD) were 3.88 Hz/ppm and 5.002 ppm, respectively, at a low HCl gas concentration (<10 ppm). In addition, the sensitivity coefficient (k*) of the *a*-PS(1.0) colloid sensor with respect to HCHO was higher than that in the case of HF because of the lower binding affinity of the former with the *a*-PS(1.0) colloids. Based on these results, highly sensitive and reproducible *a*-PS colloids could be reused as an HCl-gas-sensing platform and used as an HCl sorbent in a gas column filter.

## 1. Introduction

The corrosive white fumes of hydrogen chloride (HCl) are generated through contact with water vapor, and HCl gas is commonly expelled into the natural environment because of the combustion of plastics, i.e., polyvinyl chloride (PVC) [1]. Exposure to high concentrations of HCl gas can considerably affect the human body, including the mucous membranes, eyes, and skin. In particular, the accidental exposure of industrial employees handling gaseous products or highly concentrated mixtures to such toxic gases can result in acute respiratory diseases and asthma. Thus, simple and inexpensive gas sensors are required for periodically monitoring the trace HCl levels at workplaces. Until now, various gas-sensing materials in which a semiconductor metal oxide is used have been studied [2,3]; however, polymer-based gas-sensing materials are highly attractive for fabricating highly sensitive and selective gas systems because of the wide selection of chemical structures and simple film deposition. The development of HCl gas sensors, such as polyaniline (PANI) nanofiber [4] and nanofibrous porphyrinated polyimide (PPI) membrane [5], has been considerably investigated. Furthermore, the porphyrin derivatives in biodegradable or doped polymer [6,7], copolymer [8], or sol–gel composite [9] and TiO_2_ films [10] have been used as optical/optochemical sensors. In addition, alternative copolymerization of oligo(p-phenyleneethynylene) (OPE) has been reported with respect to fluorescent sensing systems [11]. In the case of quartz crystal microbalance (QCM)-based sensors, Matsuguchi et al. used various polymer-based materials, including morpholine-modified poly(styrene-co-chloromethyl styrene) [12], poly(acrylamide) derivatives [13], poly(N-isopropylacrylamide) (PNIPAM) nanoparticles [14,15,16], and surface-grafted PNIPAM chains [17], to detect HCl gas. However, despite the use of various functional polymers, the reusability of the coated films was low in the case of the irreversible adsorption of the copolymer [12]. Furthermore, surface grafting required precise synthesis conditions and high product reliability during device fabrication.

To overcome these limitations, we report a simple strategy to fabricate reusable HCl-gas-sensing platforms using aminated polystyrene (*a*-PS) colloids. Initially, polystyrene (PS) colloidal beads were synthesized, and their diameters were uniformly controlled. Nitrated PS colloids with various DNs were obtained, thereby controlling the volume ratio (*v*/*v*) of sulfuric acid (H_2_SO_4_) and nitric acid (HNO_3_). Then, after amination, various *a*-PS colloids were successfully manipulated. The *a*-PS colloids were spin coated on the quartz crystals (QCs). The resonant frequency (Δ*f*) measurement was performed under HCl gas flow to study the sensitivity, reusability, and selectivity of the gas-sensing platform.

## 2. Materials and Methods

### 2.1. Materials

Styrene (≥99%), polyvinylpyrrolidone (Mw ~55,000), ammonium persulfate (ACS reagent, ≥98%), and tin(II) chloride dihydrate (98%, reagent grade) were purchased from Sigma-Aldrich (St. Louis, MI, USA). The ethanol serving as the polymerization solvent (≥99.9%, absolute for analysis Emsure^®^) and that for other uses (94.5%) were purchased from Merck KGaA (Darmstadt, Germany) and Daejung Chemicals & Metals Co Ltd. (Shiheung, South Korea), respectively. Sulfuric acid (95%) and nitric acid were purchased from Duksan Pure Chemicals Co., Ltd. (Ansan, South Korea). All the chemicals were used without further purification.

### 2.2. Synthesis of the Aminated PS (a-PS) Colloidal Beads

Polyvinylpyrrolidone (PVP) (0.04 g) and styrene (8.0 g) in ethanol (88 mL) were added into a round 500 mL flask to synthesize pristine PS (*p*-PS) colloidal beads [18,19]. The solution was maintained at 70 °C in an oil bath while being stirred at 300 rpm, and polymerization began with the addition of 0.255 g of the ammonium persulfate aqueous solution (24 mL). After the reaction time of 12 h, the solution was cooled to room temperature and the PS beads were separated via centrifugation at 4000 rpm for 30 min. The *p*-PS beads were washed several times using water and ethanol through centrifugation and finally collected using a polyvinylidene fluoride (PVDF) membranous filter (pore size = ~0.45 μm, Scilab Korea Co., Ltd., Seoul, South Korea) and dried at room temperature.

0.3 g of the *p*-PS colloidal beads were added to 5 mL of nitric acid in a 20 mL vial and dispersed using a sonicator for 90 min to prepare nitrated PS (*n*-PS) colloidal beads [20]. The mixture was cooled to 0 °C using an ice bath and a solution of sulfuric acid (H_2_SO_4_)/nitric acid (HNO_2_) (*v*/*v*) was then slowly dropped into the solution. The reaction was performed for 1 h in an ice bath while being stirred at 600 rpm. After the reaction was completed, the solution that reacted with the PS colloidal beads was inserted into 50 mL of ice water in a 100 mL vial to dilute the remaining acid; next, the diluted solution was filtered using the PVDF membrane filter. After drying at room temperature, the PS beads were washed several times using water via redistribution and filtering.

Furthermore, *a*-PS colloidal beads were prepared as follows. 0.2 g of the *n*-PS beads was added to 7.5 mL of absolute ethanol in a 20 mL vial and dispersed using sonication for 90 min. Next, 3 g of tin(II) chloride dihydrate was dissolved in the solution. The reaction occurred for an hour at 70 °C while the solution was being stirred at 500 rpm. The *a*-PS colloidal beads were separated using the PVDF membrane filter, dried, and redispersed in distilled water to eliminate the residual reactants. The APS beads were separated using the membrane filter after the addition of 1 M NaOH to the solution to achieve pH 7. The washing process was repeated several times to eliminate the impurities.

### 2.3. Preparation of a-PS-Coated Quartz Crystals

250 mg of *a*-PS beads was added to a 0.2 M HCl aqueous solution and dispersed via sonication for several hours. A 50 μg *a*-PS bead dispersion solution was dropped on a gold-coated QC (9 MHz AT-cut) and spin coated at 1500 rpm for 60 s and 4000 rpm for 5 s. The *a*-PS-coated QCs were washed several times using water and dried at room temperature. Each QC was immersed in a 1 M NaOH aqueous solution for 1 h and then washed with water and dried to neutralize the amine moieties of the *a*-PS beads. All the HCl gas adsorption experiments were conducted in a stainless steel gas chamber. The QC was connected to the QCA 922 analyzer (Seiko EG&G, Chiba, Japan) and stabilized by injecting 500 sccm (cc/min) of N_2_ gas for 10 min. Then, the resonance frequency change (Δ*f*) was recorded during the HCl adsorption and desorption processes, i.e., 50 sccm of HCl gas was injected for 15 min and 500 sccm of N_2_ gas was injected for 10 min, respectively. The sensitivity factor was approximately 0.1834 Hz cm^2^ ng^−1^ in the case of 9-MHz AT-cut gold-coated QCs. A decrease of 1 Hz in the Δ*f* value was almost equal to 1.07 ng with respect to the mass loaded on the defined gold area (diameter of 5 mm, A = 0.19625 cm^2^) according to the Sauerbrey equation [21,22].

### 2.4. Characterization

The SEM images of the *p*-PS/*a*-PS beads were obtained using a field emission scanning electron microscope (FE-SEM, Hitachi S-4800, Tokyo, Japan). The samples were sputter coated with Pt for SEM analysis. The Fourier transform infrared (FT–IR) spectra were recorded using a Jasco FT/IR-4100 spectrometer equipped with a Jasco attenuated total reflectance accessory (ATR, model PR0450-S).

## 3. Results and Discussion

**Figure 1:**
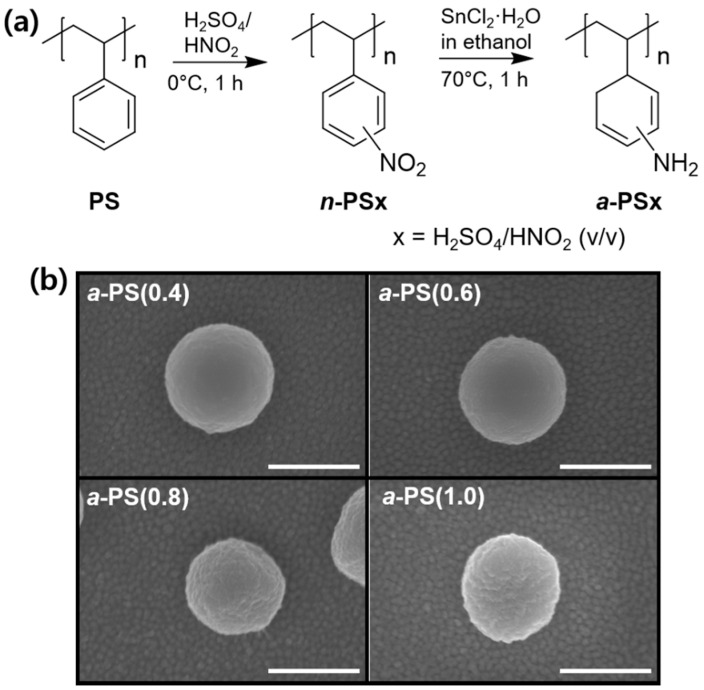
(**a**) The synthetic route of aminated polystyrene (PS) colloidal beads and (**b**) scanning electron microscope (SEM) images of four different aminated PS colloidal beads (*a*-PS(0.4, 0.6, 0.8 and 1.0)). The scale bars represent 500 nm.

**Figure 2:**
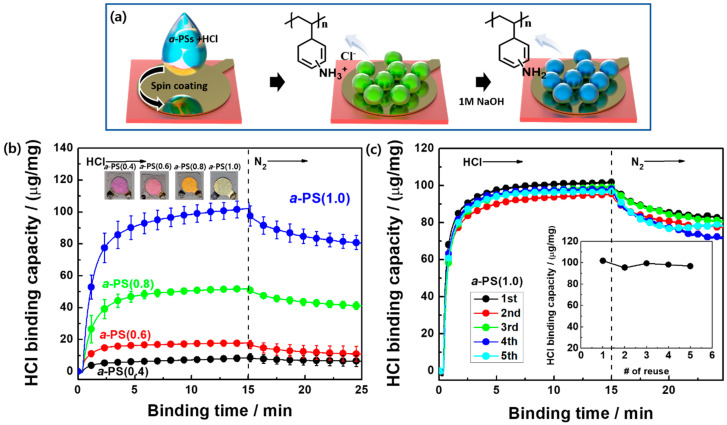
(**a**) Fabrication process of the *a*-PS colloid-spin-coated QCs, (**b**) HCl binding capacity of four different *a*-PS colloid sensors as a function of the binding time, and (**c**) HCl binding capacity of the *a*-PS(1.0) sensor as a function of the binding time in five reuses.

The PS colloidal beads were synthesized based on the synthesis method described in a previous report [18,19]. The diameter of each bead was approximately 639 ± 26 nm, as shown in Figure S1. A nitration process was performed using pristine PS (*p*-PS) colloids by controlling the volume ratio of the H_2_SO_4_/HNO_2_ mixture. The degree of nitration (DN) of the PS colloids was approximately 1 when the mixture exhibited ratios of more than 1:1, indicating that the styrene repeating units of the PS colloids were completely substituted into the nitro groups (–NO_2_). The DN obtained for each condition is summarized in Table S1. As shown in Figure S2, the FT–IR spectra showed an increase of N=O stretching peaks at 1342 and 1512 cm^−1^ as the H_2_SO_4_ volume ratio increased. After the synthesis of *n*-PS colloids, amination was sequentially performed to attach the amine group on to the styrene repeating units. The NO_2_ groups were converted to NH_2_ groups through the second step, as shown in Figure 1, and the FT–IR spectra of the *a*-PS colloids represent two distinct increasing peaks related to the amine functional groups, i.e., C–N stretching (1266 cm^−1^) and N–H bending (1620 cm^−1^), which are directly proportional to the number of nitrated styrene repeating units (Figure S3). Figure 1b shows the SEM images of four different *a*-PS colloids with controlled DAs. The shape of each single *a*-PS colloid was retained after functionalization. However, the wrinkled surface became significantly rough based on the high DA and DN, and the diameter (~567 ± 11 nm) of the *a*-PS(1.0) colloid decreased owing to the structural reorganization obtained based on the functional groups. The *a*-PS colloids were dispersed in the HCl aqueous solution to prevent colloid aggregation, spin coated on QCs, and the *a*-PS colloid-coated QCs were immersed in the NaOH solution to recover the amine groups for fabricating HCl-detecting QCM sensors (Figure 2a).

As shown in Figure S4, QCM-based HCl sensors were prepared by spin coating each *a*-PS colloid (Δ*m* = 1 μg) on a QC to measure the Δ*f* value obtained during the adsorption of the HCl gas. The Δ*f* value was measured when 100 ppm of HCl gas was introduced into the sensing vessel for a sensor operation of 15 min. N_2_ gas was flowed in for 10 min to investigate the signal reversibility. Figure 2b shows the Δ*f* value and HCl binding capacity as a function of the binding time for four types of *a*-PS colloids. *a*-PS(1.0) exhibited the highest binding capacity (102 μg/mg) and a twelve-fold higher sensing response when compared with that exhibited by the *a*-PS(0.4) colloid (8.6 μg/mg). As the DA increased, the HCl binding mass increased owing to the increased number of binding sites (Figure S5). However, the signal was not completely reversible during the 10 min N_2_ gas flow because of the chemical reaction with the amine groups on the *a*-PS colloids. Thus, the *a*-PS colloid-based QCM sensor was enabled for application in one-shot HCl detection. However, the used *a*-PS colloids can be recovered through chemical treatment. Therefore, the reusability of the HCl sensor was investigated using the *a*-PS(1.0) colloid-coated QCM sensor. After each HCl adsorption, the sensor was immersed in a 1 M NaOH aqueous solution for 1 h to eliminate the bound Cl^-^ ions and dried with gaseous N_2_; it was spin coated again on the QCs for adsorbing 100 ppm of HCl. As shown in Figure 2c, the HCl binding capacity was mostly maintained within 94% for the five HCl measurements. Thus, the gas sensors with functionalized colloidal beads could exhibit good reliability when applied in other gas-sensing systems.

**Figure 3:**
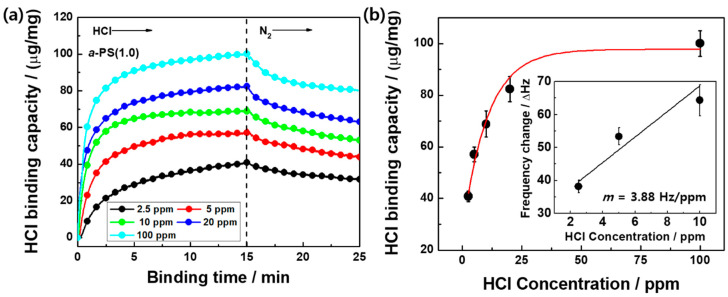
(**a**) The HCl binding capacity of the *a*-PS(1.0) sensor as a function of binding time (the process contains 15 min HCl injection and 10 min N_2_ gas flow) and (**b**) HCl binding capacity of the *a*-PS(1.0) sensor as a function of HCl concentration (from 2.5 to 100 ppm) (inset: resonant frequency change for 15 min HCl gas flow in a range of 2.5–10 ppm).

As shown in Figure 3a and Figure S6, the Δ*f* values were monitored with respect to an HCl gas concentration of 2.5–100 ppm to determine the sensitivity of the HCl-detecting *a*-PS(1.0) colloids. The Δ*f* value of the *a*-PS(1.0) colloid increased with the corresponding increase in the concentration of the HCl gas for 15 min. In addition, the HCl binding capacity was saturated at a high concentration (>40 ppm) within 15 min. Linear calibration curves were obtained at a low concentration (<10 ppm); their coefficient of determination (R^2^) was 0.806 and their sensitivities were ~3.88 Hz/ppm. The regression equations obtained from the linear calibration curves were used to calculate the limit of qualification (LOQ) and limit of detection (LOD) as LOQ = 10 (S/m) and LOD = 3.3 (S/m), respectively, where S is the standard deviation of the intercept and m is the slope of the regression line [23,24]. The LOQ and LOD values of 15.175 and 5.002 ppm, respectively, were obtained according to these equations. Moreover, adsorption isotherm was represented to investigate the interaction between HCl gas and a-Ps colloids, which indicated the distribution relationship of adsorbed HCl in an equilibrium state. As shown in Figure S7, the HCl gas adsorption followed the Langmuir isotherm model [25], in which the correlation coefficient and adsorption equilibrium constant were 0.99967 and 0.2265 L/mg.

**Figure 4:**
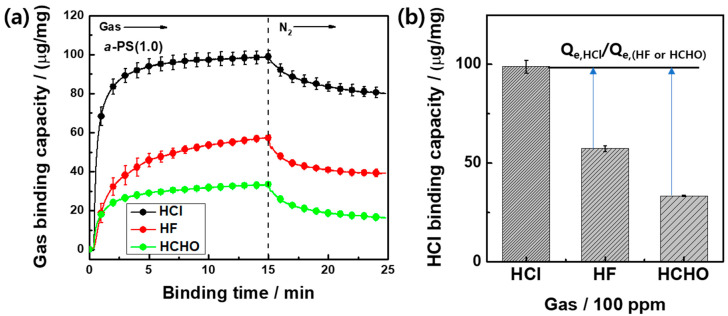
(**a**) The gas binding capacity of the *a*-PS(1.0) sensor as a function of binding time under the flow of three different gases (HCL, HF, and HCHO) at 100 ppm for a 25 min adsorption/desorption process and (**b**) the selectivity coefficient (k*) (Q_e,HCl_/Q_e,(HF or HCHO)_ of HCl with respect to other gases.

Three different gases, including HCl, were used for determining the specific selectivity, verifying the efficiency of the QCM-based HCl gas sensor. Each Δ*f* value of the 1 μg *a*-PS(1.0) sensor was measured under a 100-ppm gas flow for 15 min (Figure S8). When compared with the adsorption of the HCl gas (Δ*f* value = −92.4 ± 3.0 Hz), the sensing response of other gases were −53.5 ± 1.3 Hz for HF and −31.2 ± 0.3 Hz for HCHO corresponding to gas binding capacities of 57.3 ± 1.3 and 33.4 ± 0.3 μg/mg, respectively. As shown in Figure 4a, HCl adsorption exhibited the best binding behaviors because of strong electrostatic interaction with the amine groups; however, the HF and HCHO molecules were also able to bind with the amine groups to some extent. Furthermore, the HF molecules exhibiting strong hydrogen binding showed improved gas adsorption behaviors when compared with the sensing response of the HCHO molecules. During the desorption process under 15 min N_2_ flow, 18.9% HCl molecules were eliminated from the *a*-PS(1.0) colloids. However, the desorption percentages of the HF and HCHO molecules decreased to 32.6% and 51.4%, respectively, because of the low binding affinity. As shown in Figure 4b, the selectivity coefficient (k*) (i.e., the ratio of the Q_e,HCl_/Q_e,(HF or HCHO)_) of the *a*-PS(1.0) colloid sensors was 1.73 for HF and 2.96 for HCHO.

## 4. Conclusions

In this study, we developed QCM-based HCl sensors using *a*-PS colloidal beads. Their sensing response (Δ*f*) varied depending on the DA. The *a*-PS(1.0) colloids exhibited the highest binding capacity (102 μg/mg) and reasonable reusability. At low concentrations (<10 ppm), the HCl adsorption on the *a*-PS(1.0) colloids resulted in a sensitivity of 3.88 Hz/ppm and an LOD value of 5.002 ppm. Furthermore, the k* value of the *a*-PS(1.0) colloidal sensor with respect to HCHO was higher than that obtained with respect to HF because of the lower binding affinity of HCHO to the *a*-PS(1.0) colloids. Based on these results, highly sensitive and reproducible *a*-PS colloids could be reused for HCl gas sensing and potentially applied as HCl sorbents in a gas column filter.

## Supplementary Materials

The following are available online at https://www.mdpi.com/2073-4360/12/7/1591/s1: Figure S1: SEM image of the synthesized PS colloidal beads; Figure S2: FT–IR spectra of the n-PS and a-PS colloids; Figure S3: HCl binding capacity on various a-PS colloids under 100-ppm HCl gas flow and the relation of HCl binding capacity and degree of amination as a function of sulfuric ratio.
