# Supplementary material for: QCM-Based HCl Gas Sensors Using Spin-Coated Aminated Polystyrene Colloids
Source: Polymers (Basel). 2020 Jul 17;12(7):1591. doi: 10.3390/polym12071591 (PMC7408365; doi:10.3390/polym12071591)
Supplement: Supplementary file 1 [file polymers-12-01591-s001.pdf]

Supporting Information

QCM-Based HCl Gas Sensors Using Spin-Coated Aminated Polystyrene Colloids

Young-Jae Jin[1] and Jinyoung Park[1,2,*]

[1]*School of Applied Chemical Engineering, Kyungpook National University, 80 Daehak-ro, Buk-gu, Daegu 41566, Republic of Korea*

[2]*Department of Polymer Science & Engineering, Kyungpook National University, 80 Daehak-ro, Buk-gu, Daegu 41566, Republic of Korea*

***** Correspondence: jinpark@knu.ac.kr (J.P.);
Tel.: +82-53-950-5624 (J.P.)

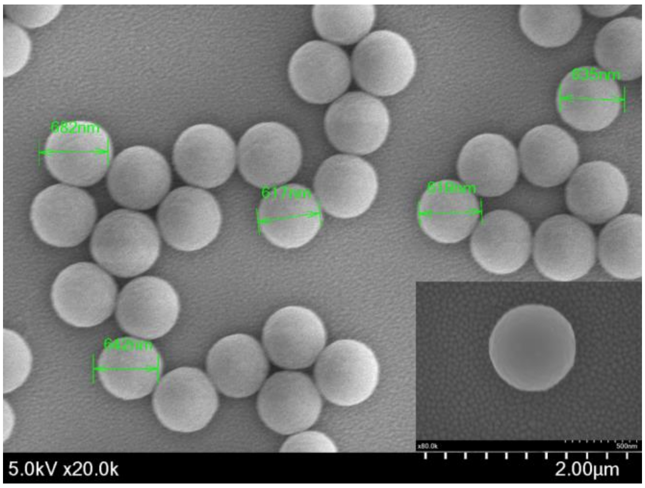

Figure S1. SEM images of the pristine PS colloidal beads.

Table S1. Nitration of PS colloidal beads using a mixture solution of H_2SO_4 and HNO_3 (v/v).

Samples	H_2SO_4/HNO_3 (v/v)	Input (g)	Yield (g)	Yield ratio	Degree of nitration (DN)
n-PS(0.4)	0.4	0.2	0.205	1.025	0.05869
n-PS(0.6)	0.6	0.2	0.223	1.115	0.26995
n-PS(0.8)	0.8	0.2	0.259	1.295	0.69249
n-PS(1.0)	1.0	0.2	0.287	1.435	1.02113
n-PS(1.2)	1.2	0.2	0.285	1.425	0.99765

a) Styrene (repeating unit: 105.18 g/mol) and nitrated styrene (repeating unit: 150.06 g/mol)

b) Yield ratio (g/g) = 150.06/105.18 = 1.426 if the substitution ratio is 1.

c) Degree of nitration (DN) = (yield ratio − 1)/0.426

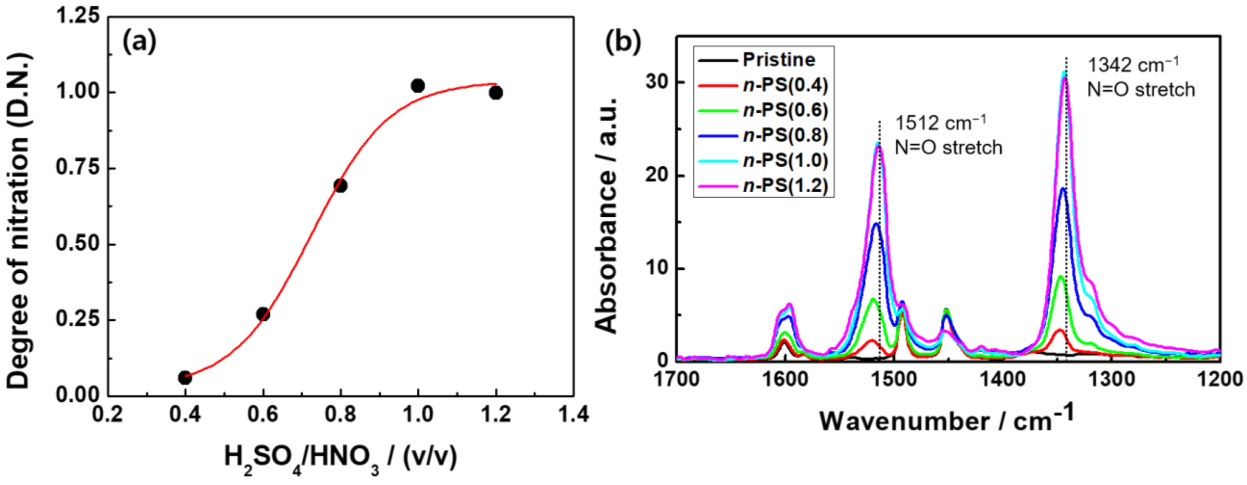

Figure S2. (a) Degree of nitration (DN) as a function of H_2SO_4/HNO_3 (v/v) and **(b)** FT–IR spectra of the nitrated PS (*n*-PS) colloids with five different DN values, including pristine PS (*p*-PS) colloids.

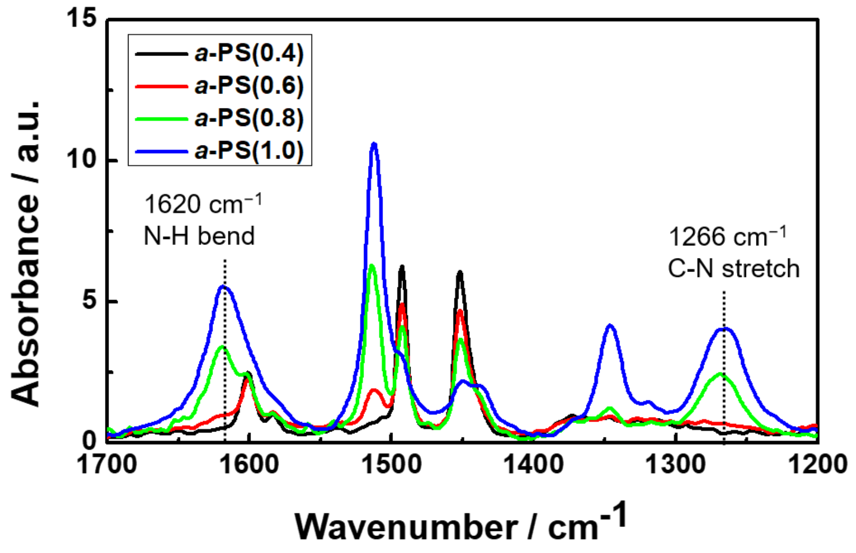

Figure S3. FT–IR spectra of the aminated PS (*a*-PS) colloids with four different DN values.

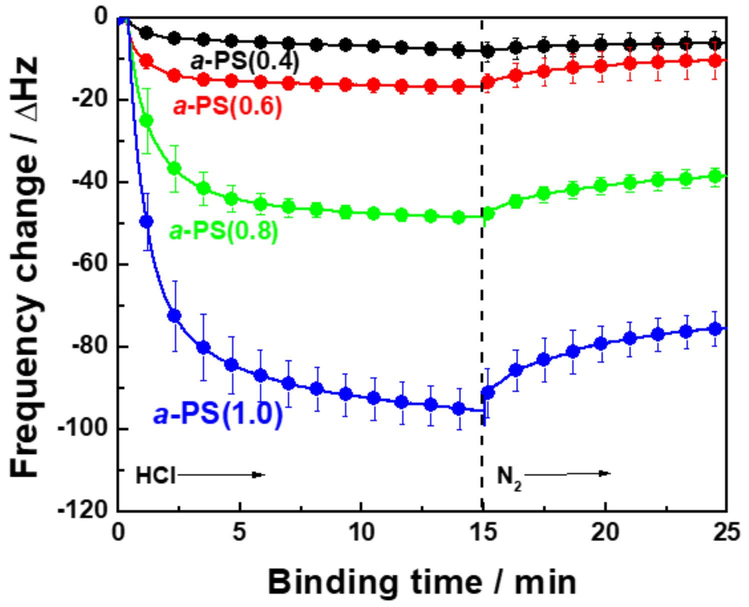

Figure S4. Frequency change (Δf) as a function of time on four different *a*-PS colloid sensors (Δm = 1 μg) in case of a 100-ppm HCl gas flow for the 15-min adsorption and 10-min desorption processes (n = 3).

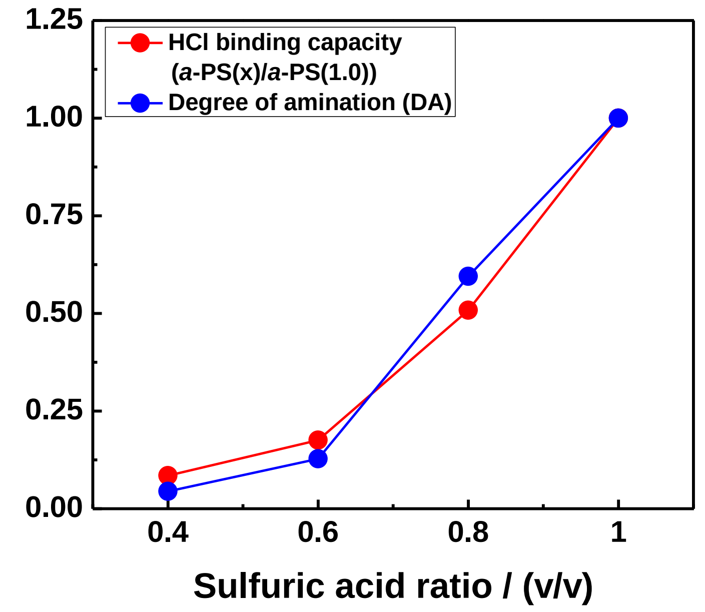

Figure S5. The correlation of HCl binding capacity with degree of amination. The ratio of HCl binding capacity was calculated from *a*-PS(x)/*a*-PS(1.0) and the ratio of degree of amination was obtained by calculating ($A_{a\text{-PS(x)}}$-$A_{pristine}$)/ ($A_{a\text{-PS(1.0)}}$-$A_{pristine}$) from absorbance intensity of FT-IR at a peak of 1620 cm^{-1}.

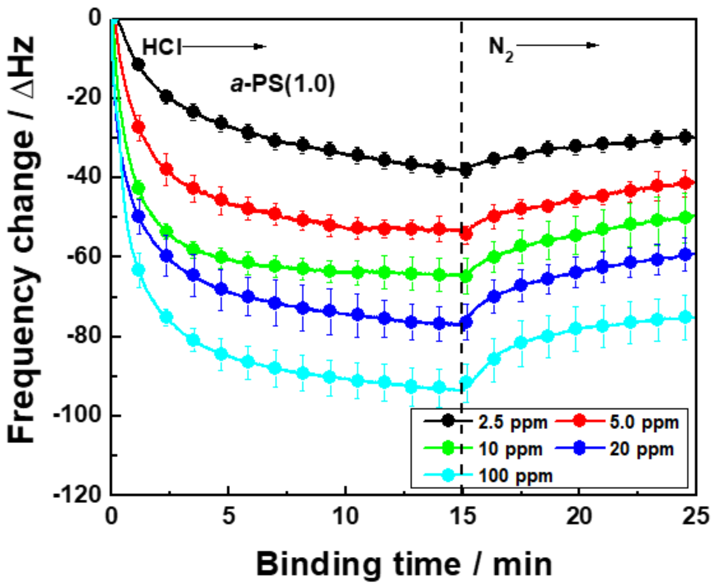

Figure S6. Frequency change (Δ*f*) as a function of time on the *a*-PS(1.0) colloid sensor (Δ*m* = 1 μg) under an initial HCl gas flow (2.5–100 ppm) (n = 3) for the 15-min adsorption and 10-min desorption processes (n = 3).

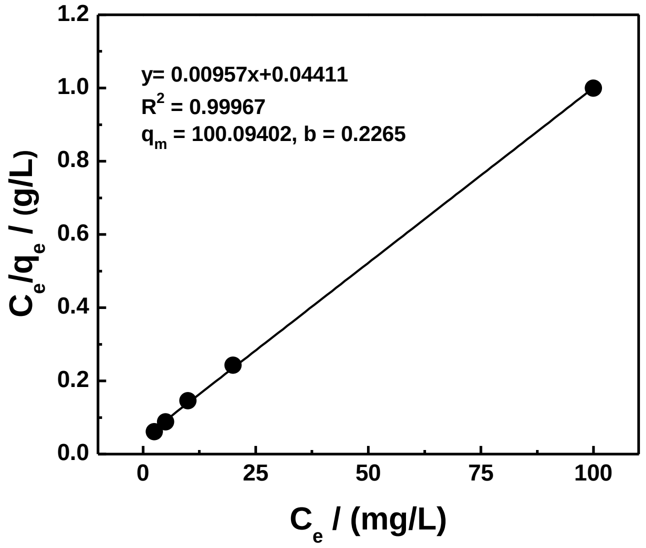

Figure S7. Langmuir isotherm for the adsorption of *a*-PS(1.0). Langmuir isotherm equation ($C_e/Q_e = 1/(bQ_m) + C_e/Q_m$) was used, where q_e (mg/g) and q_m (mg/g) are the maximum and equilibrium adsorption capacity of the HCl gas, respectively; b (L/mg) is the adsorption equilibrium constant; and Ce (mg/L) is the equilibrium concentration (mg/L).

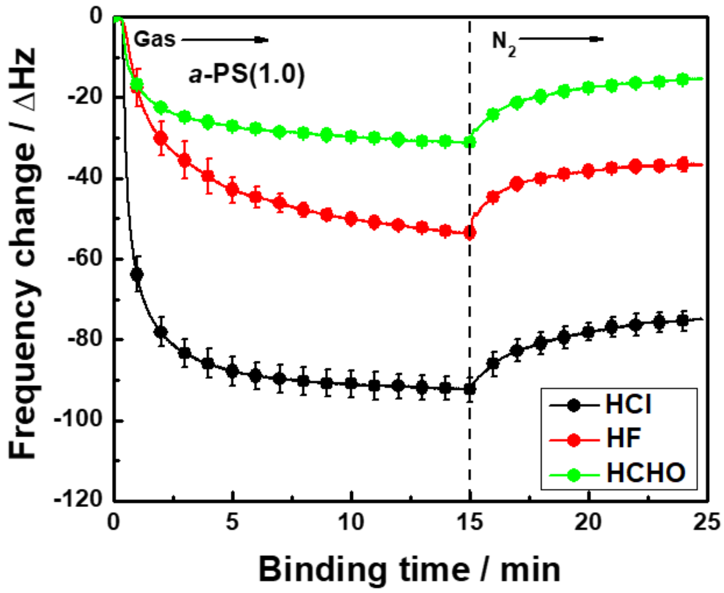

Figure S8. Frequency change (Δf) as a function of time on the *a*-PS colloid sensor (Δm = 1 μg) for the HCl, HF, and HCHO gases at a fixed concentration (100 ppm) for the 15-min adsorption and 10-min desorption processes (n = 3).
